# Healthcare Professionals’ Perceptions About Medical Cannabis in Greece: A Qualitative Study

**DOI:** 10.3390/jmahp13020013

**Published:** 2025-04-02

**Authors:** Christos Ntais, Yioula Melanthiou, Michael A. Talias

**Affiliations:** 1 Healthcare Management Program, School of Economics & Management, Open University of Cyprus, Nicosia 2220, Cyprus; michael.talias@ouc.ac.cy; 2 Epidemiology Program, School of Science and Technology, Hellenic Open University, 26335 Patras, Greece; 3 Marketing Department, School of Business, University of Nicosia, Nicosia 2417, Cyprus; 4 Department of Communication and Marketing, Faculty of Communication and Media Studies, Cyprus University of Technology, Limassol 3036, Cyprus; yioula.melanthiou@cut.ac.cy

**Keywords:** medical cannabis, scientific evidence, social stigma, regulatory barriers, communication strategies, health policy, market access

## Abstract

Background: Medical cannabis continues to generate interest as a potential therapeutic option, yet its acceptance in clinical practice faces challenges, including regulatory barriers, social stigma, and gaps in scientific evidence. Methods: This study explores the perspectives of Greek medical doctors and pharmacists on medical cannabis—key stakeholders in its clinical application—through semi-structured interviews with 12 participants from each profession. Results: Medical doctors and pharmacists expressed a range of views on medical cannabis, with many acknowledging its potential while emphasizing the need for rigorous, disease-specific research. Medical doctors highlighted the lack of consistent clinical trials, concerns about drug interactions, and the fine line between medical use and misuse. Pharmacists echoed these concerns, citing regulatory inconsistencies and the need for standardized dosing. Both groups agreed that social stigma and misinformation hinder cannabis adoption, advocating for targeted education and transparent research communication. Participants indicated that regulatory barriers also pose challenges, with calls for harmonized policies and phased market entry approaches. Effective communication strategies, including digital outreach and clear messaging, were suggested to differentiate medical cannabis from recreational use and improve trust among healthcare providers and patients. Participants also highlighted the urgent need for collaboration between policymakers, researchers, and healthcare professionals to establish medical cannabis as a credible therapeutic option. Conclusion: The insights gained provide actionable recommendations to bridge existing gaps and emphasize the need for a responsible, evidence-based approach to the acceptance of medical cannabis as a therapeutic option.

## 1. Introduction

The potential benefits of medical cannabis are gaining attention, particularly in the management of conditions such as childhood epilepsy and spasticity in multiple sclerosis [1]. A benefit of its use has been demonstrated in relieving symptoms such as chemotherapy-induced nausea and vomiting [2] and chronic non-cancer pain [3]. However, significant challenges remain in positioning cannabis as a legitimate medical product.

A major challenge is the lack of standardization in research and regulation. Across regions, inconsistent legal frameworks complicate prescribing practices and hinder the development of clear clinical guidelines. For instance, while some jurisdictions have embraced cannabis for medical use, others maintain strict prohibitions, creating a fragmented landscape [4,5]. This regulatory patchwork also limits access for patients who could benefit from cannabis-based therapies.

Another barrier is the social stigma associated with cannabis, rooted in its historical criminalization and association with recreational use. This stigma not only affects public perceptions but also influences healthcare providers’ willingness to recommend or prescribe cannabis [6,7,8]. Addressing these cultural and professional biases is essential for considering cannabis as a therapeutic option in carefully selected patient populations [9,10].

Moreover, the scientific evidence supporting medical cannabis, while promising, remains incomplete. Existing studies often lack the methodological rigour necessary to establish definitive conclusions about its safety and efficacy. Previous research has highlighted the need for detailed studies into dosing, administration methods, and potential drug interactions to guide safe and effective use [11,12].

Greece has recently taken legislative steps toward regulating medical cannabis with the aim of creating a controlled framework for its cultivation, distribution, and prescription. The legal status of medical cannabis varies depending on whether the formulation contains THC (tetrahydrocannabinol) or CBD (cannabidiol). THC-containing medical cannabis was legalized for medicinal use in 2017. In 2018, the country lifted restrictions on the cultivation and production of cannabis for medical purposes, allowing licensed companies to grow and process cannabis with high THC content. However, access to medical cannabis with THC is strictly regulated. Patients can obtain it only with a physician’s prescription, and it is available exclusively through a bureaucratic procedure. On the other hand, CBD products with a THC concentration below 0.2% are fully legal and widely available. CBD is not classified as a narcotic in Greece, and consumers can purchase it without a prescription. Various CBD products, such as oils, capsules, and dried flower, are sold in pharmacies, health stores, and online shops.

This qualitative study examines the perspectives of Greek healthcare professionals regarding medical cannabis, focusing on their opinions about its therapeutic value, barriers to its adoption, and recommendations for future research and policy development. By leveraging insights from semi-structured interviews with 12 medical doctors and 12 pharmacists, a nuanced understanding of the opportunities and challenges in advancing medical cannabis as a therapeutic option is provided. By synthesizing their views, this study aims to offer actionable recommendations for policymakers, healthcare providers, and industry stakeholders to design strategies that balance patient access, medical credibility, and public health concerns, ultimately paving the way for the responsible acceptance of medical cannabis in clinical practice.

## 2. Methods

### 2.1. Study Design

This qualitative study utilized a semi-structured interview approach to capture in-depth perspectives on medical cannabis from healthcare professionals. The design was chosen to allow flexibility in exploring participants’ experiences, beliefs, and concerns, ensuring rich and detailed data collection. The study adopted an inductive approach, employing content analysis to identify recurring patterns and themes.

### 2.2. Participants

Participants included 12 medical doctors and 12 pharmacists of Greek nationality, purposively sampled based on their professional experience with medical cannabis or related therapeutic fields. Medical doctors were selected based on their medical specialty, ensuring relevance to the listed indications for medical cannabis in Greece. Inclusion criteria required participants to have at least five years of professional experience in their respective fields and to be actively engaged in patient care or pharmaceutical practice. Participants were recruited through professional networks and scientific conferences, ensuring a diverse representation of roles and institutions.

### 2.3. Data Collection

Data were collected through one-on-one semi-structured interviews conducted in person or via video conferencing. Interviews were conducted between October 2022 and March 2023, lasting approximately 45–60 minutes each. To ensure consistency in the topics covered, a semi-structured interview guide was developed based on the study objectives and existing literature on medical cannabis (Table 1). Key topics included: perceptions of cannabis as a therapeutic option, barriers to its adoption, unmet research needs, and policy recommendations for advancing its medical use. Interviews were audio-recorded with participants’ consent and transcribed verbatim for analysis. Field notes were also taken to capture non-verbal cues and contextual information.

### 2.4. Data Analysis

Content analysis was employed to identify and interpret recurring patterns and nuanced themes in the data. Following Braun and Clarke’s [13] framework, transcripts were reviewed and coded independently by the researchers. The initial codes were grouped into broader themes, which were refined through iterative discussions among the research team. Discrepancies in coding were resolved through consensus to enhance the credibility of the findings. NVivo 14 software was utilized to assist with data management and ensure a rigorous analytical process.

### 2.5. Ethical Considerations

Ethical approval for the study was obtained from the University of Nicosia Research Ethics Committee (Ref. No UREC/2022/14, dated 19 July 2022), ensuring compliance with established guidelines for research involving human participants. Informed consent was obtained from all participants before the interviews, with assurances of confidentiality, anonymity, and the right to withdraw at any time. To protect participants’ identities, participants were assigned aliases (MD1-MD12 for medical doctors; P1-P12 for pharmacists). Data were securely stored and accessible only to the research team to maintain privacy and confidentiality.

## 3. Results

### 3.1. Sample Characteristics

Medical doctors: The sample consisted of six males and six females, aged 41 to 59 years (mean = 49.2 years). They represented three specialties—oncology (*n* = 5), neurology (*n* = 5), and pain management (*n* = 2)—that frequently encounter patients who may use cannabinoids for medical purposes based on evidence of their benefits for the conditions they treat. Their average professional experience was 16.3 years. All medical doctors were employed in hospitals in Athens, Greece.

Pharmacists: The sample included seven males and five females, aged 32 to 61 years (mean = 45.8 years). They worked in either private pharmacies (*n* = 10) or hospital pharmacies (*n* = 2) in Athens, Greece, with an average of 17.2 years of professional experience.

### 3.2. Thematic Axes

#### 3.2.1. Perceptions of Medical Cannabis as a Therapeutic Option

Medical doctors expressed a spectrum of opinions. While many acknowledged cannabis’ potential, they often emphasized the need for robust, disease-specific research to establish its credibility. MD1 commented, “Cannabis is promising, but without comprehensive clinical trials, it remains difficult to confidently recommend it”. Similarly, MD4 stated, “Its therapeutic potential is clear in some areas, but we lack consistent, high-quality evidence for widespread use”. MD8 added a hopeful note, “If the safety and efficacy of cannabis are validated for more indications, it could revolutionize pain management”. On the other hand, MD9 noted skepticism, stating, “We’re far from understanding its full therapeutic potential and over-reliance on anecdotal evidence is concerning”.

Medical doctors also highlighted the complexity of cannabis’ role in medicine. MD5 remarked, “The interaction of cannabinoids with existing medications remains underexplored, and this limits our ability to integrate it into standard treatment protocols”. MD11 expressed a similar sentiment, “There’s a fine line between medical use and misuse, which makes it even more important to establish clear, evidence-based guidelines”.

Pharmacists were similarly divided. P3 described cannabis as a “well-developed therapeutic alternative”, citing its efficacy in conditions such as migraine and muscular pain. P5 added, “I’ve seen significant benefits in patients with pain and anxiety”. In contrast, P8 expressed concerns about regulatory inconsistencies, stating, “The safety profile is still uncertain, and more research is essential to build trust among both patients and healthcare professionals, especially pharmacists who act as critical gatekeepers in the dispensing process”. P11 highlighted, “While its benefits are clear for some patients, there’s still a gap in standardized dosing guidelines”.

Pharmacists also pointed out social barriers. P7 explained, “The lingering stigma around cannabis affects how it’s perceived by both healthcare providers and patients. We need more education to shift these perceptions”. P2 added, “Patients are often hesitant to ask about cannabis, even when it might be a viable option. This reflects the need for normalization through education and advocacy”.

#### 3.2.2. The Need for Rigorous Research

Both groups emphasized the necessity of further research to validate medical cannabis’ safety and efficacy. P7 underscored this, stating, “Longer, disease-specific research is essential to establish a reliable therapeutic profile”. MD3 highlighted the importance of isolating active compounds, remarking, “Targeted studies will help us move beyond anecdotal evidence and establish cannabis as a mainstream treatment”. MD10 added, “The complex interplay of cannabis compounds necessitates multidisciplinary research to unlock its full potential”. Similarly, P8 suggested, “Interdisciplinary research can unravel the complexities of cannabinoids and their interactions with the human body”. Finally, P9 noted, “The complexity of cannabis’ active compounds necessitates detailed clinical trials to ensure accurate dosing and efficacy. Research must also address interactions with other medications”.

Both groups also agreed on the importance of transparency in research findings. MD2 stated, “Openly sharing data, whether positive or negative, will help the field move forward responsibly”. P6 concurred, “We need to ensure that research outcomes are communicated effectively to healthcare professionals and the public to build trust”.

#### 3.2.3. Addressing Stigma Through Education

Medical doctors highlighted social stigma as a major barrier. MD2 advocated for targeted educational campaigns, stating, “Patient testimonials and real-world case studies can be powerful tools to humanize cannabis and showcase its benefits”. MD3 pointed out, “Educational campaigns can demystify cannabis and help patients feel more comfortable discussing it with their providers”. MD7 noted, “Physicians need accessible, evidence-based information to confidently recommend cannabis to their patients without fear of judgment”. MD12 suggested, “Public endorsements from respected medical organizations could be instrumental in changing perceptions”. Medical doctors also discussed the need for clarity in messaging. MD6 stated, “We need to separate the concept of medical cannabis from recreational use clearly, so patients understand the difference”.

Pharmacists emphasized the role of factual, reliable information in dispelling misconceptions. P1 stated, “Scientific data and public testimonials can demonstrate the medical potential of cannabis and counter negative stereotypes”. P6 added, “Involving healthcare professionals as advocates will lend credibility to educational efforts and help bridge gaps in perception”. P4 proposed, “Case studies presented at conferences or published in journals would highlight the therapeutic possibilities and reduce stigma”. P10 remarked, “Pharmacists need access to updated guidelines and training modules to navigate conversations with patients effectively”.

Pharmacists also noted the importance of addressing generational gaps in perception. P8 commented, “Older patients often associate cannabis with illicit use. Educating this demographic requires a sensitive, fact-based approach”. P10 added, “Bridging the gap between traditional and modern medicine can help overcome resistance among certain patient groups”.

#### 3.2.4. Overcoming Regulatory Barriers to Market Entry

Inconsistent regulatory frameworks were identified as a significant barrier to adoption. Participants highlighted the need for harmonized policies that establish clear guidelines for prescribing, dispensing, and using medical cannabis. P11 suggested, “Standardized dosing guidelines and labeling requirements can reduce confusion and build trust among both healthcare providers and patients”.

Pharmacists proposed the adoption of phased approaches to market entry in regions with progressive regulations, allowing pilot programs to gather initial data and refine strategies before broader implementation. P11 suggested, “Pilot programs in supportive jurisdictions could pave the way for broader acceptance and provide valuable data”. Medical doctors stressed collaboration with regulatory bodies, with MD10 commenting, “Close partnerships with policymakers are essential for ensuring compliance and building trust among stakeholders”. MD6 remarked, “Providing regulators with evidence-based research and involving them in educational campaigns can smooth the path to market entry”. Finally, MD9 emphasized, “Policymakers need to collaborate with healthcare professionals to ensure regulations are practical and evidence-based”.

#### 3.2.5. Communication Strategies for Improved Adoption

Both groups agreed on the importance of establishing a clear communication strategy. P4 explained, “Medical cannabis needs a distinct identity that separates it from recreational use. Clear messaging is key”. MD8 emphasized the need for products tailored to specific medical conditions, stating, “Transparent communication about benefits and limitations will help build trust”. P2 suggested, “Linking cannabis products to specific, validated therapeutic outcomes will drive acceptance”. Similarly, MD9 added, “Effective communication should highlight the conditions where cannabis has shown promise and its safety profile, backed by credible evidence”. P5 further emphasized, “Communication should focus on safety, efficacy, and consistency. These are the attributes that will resonate with both healthcare providers and patients”. MD4 added, “A robust communication strategy will also include clear dosing guidelines and validated research outcomes to support medical claims”.

Pharmacists also suggested leveraging digital tools, which offer unique opportunities for outreach and education, particularly for younger demographics who are more open to alternative therapies. P3 stated, “Online platforms can effectively target both patients and healthcare providers, offering resources and testimonials to support adoption”. P7 added, “A combination of traditional and digital marketing will ensure the message reaches diverse audiences”. Finally, P3 suggested, “Social media campaigns and online resources can effectively communicate the potential of medical cannabis while addressing misconceptions”.

## 4. Discussion

This study highlights the intricate interplay between evidence, education, and social perceptions in advancing medical cannabis as a therapeutic option. While there is a shared recognition of its potential benefits, the insights provided by medical doctors and pharmacists underscore the nuanced challenges that must be addressed for its responsible acceptance in clinical practice.

The findings of this study align with existing literature on the polarized perspectives surrounding medical cannabis. Physicians’ skepticism regarding medical cannabis is well-documented in the literature particularly in the context of its severe adverse effects, potential misuse, the high placebo response, and positive media attention in cannabis trials [14,15]. A study focusing on New Zealand physicians identified limited clinical evidence as the principal barrier to prescribing medicinal cannabis [16]. Additional concerns included a perceived lack of knowledge about medicinal cannabis, apprehensions over professional reputation, social stigma, and the cost of products [16]. Similarly, Hachem et al. [17] indicate that healthcare practitioners often feel unprepared to prescribe medical cannabis due to insufficient clinical research and uncertainties about safe and effective administration. The study suggests that this reluctance is compounded by a lack of formal education on cannabis in medical curricula, leading to hesitancy in engaging with cannabis-based treatments [17].

Pharmacists also exhibited diverse perspectives on medical cannabis, reflecting their critical role as intermediaries between patients and prescribers, aligning with previous research. A recent literature review found that while pharmacists are generally comfortable dispensing medical cannabis, they express a need for further education to ensure safe and effective patient care [18]. In addition, pharmacists are frequently at the forefront of patient education on cannabis. Dattani and Mohr [19] indicated that pharmacists play a pivotal role in counseling patients about dosing, potential drug interactions, and the safe use of cannabis products, thereby enhancing patient acceptance and adherence to cannabis-based treatments.

One of the most pressing concerns among participants was the lack of robust, disease-specific evidence to guide medical cannabis acceptance in clinical practice. Our study’s emphasis on addressing evidence gaps aligns with existing literature that cites the scarcity of high-quality clinical trials as a limiting factor. A recent systematic review emphasizes that reliable evidence is limited, particularly concerning cannabidiol (CBD), with only 6.7% of CBD trials meeting high-quality standards [20]. The review underscores the necessity for more rigorous studies to establish clear clinical guidelines for medical cannabis use. Similarly, Simei et al. [21] discuss the challenges in developing cannabinoid-based drugs, noting that the global interest in therapeutic uses of CBD is outpacing the scientific evidence supporting its safety and efficacy. They call for more basic and clinical research adhering to regulatory guidelines to obtain approval for broader clinical indications. Finally, Senator et al. [22] analyzed 23 contemporary systematic reviews on the effectiveness and safety of medical cannabis. They found that the existing body of work often lacks the methodological rigor necessary to draw definitive conclusions, highlighting the need for more targeted and high-quality clinical trials.

Additionally, the emphasis on collaborative research aligns with the growing recognition that interdisciplinary efforts are necessary to address the complexities of medical cannabis, including its pharmacodynamics and potential interactions with existing medications [23]. This underscores the importance of partnerships between academia, industry, and policymakers, which can expedite the generation of reliable data and foster trust among stakeholders.

Social stigma emerged as a significant barrier to the broader acceptance of medical cannabis. Many participants emphasized the need for targeted educational initiatives aimed at normalizing cannabis use in medical contexts. The role of education in combating stigma and enhancing healthcare provider confidence is similarly reflected in prior studies. Literature consistently advocates for targeted educational interventions to bridge knowledge gaps and address misconceptions, which our study also identified as critical. Kruger et al. [24] highlight that many healthcare providers report low levels of knowledge about medical cannabis, which can hinder patient-provider discussions. They emphasize the importance of comprehensive education and training for healthcare professionals to facilitate open and informed conversations with patients regarding medical cannabis use. Additionally, Schuhmacher et al. [25] suggest that healthcare providers have significant knowledge gaps in authorizing medical cannabis, which limited their practice competence and confidence in this area. They recommend developing practice guidelines and educational resources to support primary care providers in medical cannabis authorization within the healthcare system. Both studies underscore the need for enhanced training and education among healthcare providers to normalize and improve discussions about medical cannabis with patients, echoing the perspectives of participants in our study.

Regulatory challenges emerged as a significant barrier to medical cannabis adoption. Our study’s findings on the fragmented nature of regulations and the need for harmonized policies are consistent with calls for global and national frameworks that establish clear guidelines for prescribing and dispensing. Mead [26] discusses the complex legal and regulatory frameworks surrounding cannabis in the United States, highlighting the challenges posed by inconsistent regulations at both federal and state levels. The study emphasizes the necessity for a cohesive regulatory approach to facilitate medical cannabis adoption. In addition, de Souza et al. [27] provide an overview of various regulatory frameworks for medical cannabis worldwide, focusing on the Brazilian scenario. The study highlights the disparities in regulations and advocates for harmonized policies to ensure safe and effective access to medical cannabis.

Moreover, a structured regulatory framework for medical cannabis could enhance its acceptance among healthcare professionals by providing clear guidelines and quality control measures. A scoping review identified that the need for more knowledge, advice, and empirical/clinical evidence is a significant facilitator in the implementation of medical cannabis regulations [4]. Countries with well-defined medical cannabis regulations have seen higher acceptance rates among physicians and pharmacists. For instance, Germany’s accommodative framework allows patients to access cannabis through established programs, contributing to the greater inclusion of medical cannabis in clinical practice [28]. These examples illustrate that comprehensive regulatory frameworks could bridge the gap between medical acceptance and patient demand for cannabis-based treatments.

Finally, our study highlights the importance of communication strategies that focus on safety, efficacy, and transparency in increasing the legitimacy of medical cannabis. A recent study analyzing Instagram posts related to cannabinoid use found that educational content emphasizing safety and efficacy garnered significant engagement, indicating public interest in transparent information about medical cannabis [29]. Our study’s participants recommended communication initiatives that clearly distinguish medical cannabis from its recreational counterpart. Similarly, Cheng et al. [30] indicated that healthcare professionals support the use of medical cannabis for chronic pain management but emphasize the need for clear guidelines and education to differentiate its medical applications from recreational use. Additionally, our study’s participants supported using testimonials from patients and healthcare providers to humanize medical cannabis and highlight its real-world benefits. A systematic review found that physicians experienced in prescribing medical cannabis were more convinced of its benefits and less concerned about adverse effects than non-experienced physicians, suggesting that sharing their positive experiences could enhance acceptance [31].

This research is subject to some limitations. First, the use of a non-random sampling method may limit the generalizability of the findings, as participants were purposively selected based on their professional experience with medical cannabis. Therefore, their responses are unlikely to be representative of physicians in general, as the participants are likely much more knowledgeable about and open to the medical use of cannabis than the broader physician population. Second, while the sample size was relatively small, practical research suggests that a sample of 12 participants may be sufficient to reach data saturation within a relatively homogeneous population [32]. However, a larger sample could have provided additional nuances. Finally, the study focused exclusively on the perspectives of medical doctors and pharmacists, excluding other key stakeholders such as patients, policymakers, and representatives from the pharmaceutical industry. Including these perspectives could have further enriched our findings and provided a more comprehensive understanding of the challenges and opportunities surrounding medical cannabis acceptance in clinical practice.

## 5. Recommendations for Future Research

Future research should adopt a multi-dimensional approach to expand the understanding of medical cannabis and balance its risks and benefits. Studies should prioritize exploring the perspectives of patients and policymakers, as these stakeholders provide critical insights that complement the views of healthcare professionals. Understanding patient attitudes can highlight barriers to acceptance, such as stigma or lack of knowledge, while policymakers’ perspectives can inform regulatory frameworks that balance accessibility with safety.

Longitudinal studies are necessary to evaluate the sustained impact of educational and communication initiatives aimed at normalizing medical cannabis. These analyses can measure changes in healthcare professionals’ prescribing behaviors, patient acceptance, and public perception over time, offering evidence-based recommendations for scaling successful strategies.

Regional variations in the adoption of medical cannabis must also be investigated. By examining the socio-cultural, economic, and regulatory factors that influence acceptance in different areas, tailored strategies can be developed to address specific challenges. For instance, comparative studies between regions with advanced cannabis programs and those with restrictive policies could reveal best practices for implementation and adaptation.

Finally, research should explore interdisciplinary collaborations to address gaps in knowledge about the safety and efficacy of medical cannabis. Such efforts can accelerate the development of standardized guidelines and foster trust among stakeholders. By addressing these research priorities, a comprehensive roadmap for the responsible evaluation of medical cannabis can be created, benefiting patients, providers, and society at large.

## 6. Conclusions

The evidence-based inclusion of medical cannabis in clinical practice represents a transformative opportunity to address unmet therapeutic needs while navigating complex social and scientific challenges. This study underscores the importance of leveraging the perspectives of both medical doctors and pharmacists to inform strategies that foster responsible acceptance, improve education, and enhance market trust.

Achieving this goal requires a multi-faceted approach that prioritizes rigorous research, comprehensive education, and consistent regulatory frameworks. Research initiatives should aim to address critical gaps in evidence, particularly in understanding cannabis’ efficacy for specific health conditions. High-quality clinical trials are essential to establish robust evidence for the efficacy and safety of medical cannabis. To facilitate this research, regulatory authorities must work toward reducing operational barriers to clinical trials of medical cannabis. This includes providing non-industry research funding, ensuring access to appropriate study products, and easing regulatory restrictions such as prohibitive dispensing regulations. Addressing these challenges is crucial to advancing scientific understanding and informing evidence-based medical practice. On the other hand, educational efforts must focus on dispelling stigma, equipping healthcare professionals with the tools and confidence to recommend cannabis when appropriate, and ensuring patients feel informed and supported.

Policymakers and industry stakeholders have an essential role in establishing standardized regulations that build trust among all parties. Clear labeling, dosing guidelines, and well-communicated safety profiles can help position medical cannabis as a credible therapeutic option. Furthermore, targeted communication strategies that emphasize patient outcomes and align with scientific evidence can counteract misconceptions and promote wider adoption.

The findings from this study highlight that the path to mainstream acceptance of medical cannabis is one of collaboration and persistence. By addressing these challenges systematically, medical cannabis has the potential to transition from a promising alternative to an established therapeutic option, improving patient outcomes.

## Figures and Tables

**Table 1:** Topic Guide.

	Question
1	Can you describe your professional background and experience in the healthcare field?
2	Have you encountered medical cannabis in your practice? If so, in what capacity?
3	What is your general perception of medical cannabis as a therapeutic option?
4	What are your views on the potential benefits of medical cannabis?
5	Do you believe there is sufficient clinical evidence to support its use? Why or why not?
6	In your opinion, what are the most promising health conditions for medical cannabis treatment?
7	What concerns do you have about its integration into medical practice?
8	How do you think medical cannabis compares to existing pharmaceutical treatments?
9	What type of research do you believe is most needed to validate the use of medical cannabis?
10	Do you think current studies provide sufficient evidence for widespread use? If not, what is lacking?
11	How important is interdisciplinary research in understanding the pharmaceutical effects of cannabis?
12	How can transparency in research findings improve medical cannabis acceptance?
13	Do you believe there is a stigma associated with medical cannabis? Why or why not?
14	How does this stigma impact healthcare providers and patients?
15	What role do you think education plays in changing perceptions of medical cannabis?
16	What strategies do you suggest to provide more accurate information to healthcare providers and patients?
17	How can medical institutions and organizations contribute to reducing stigma?
18	What regulatory challenges do you see in prescribing and dispensing medical cannabis?
19	How do inconsistent regulations affect medical cannabis adoption?
20	What steps should policymakers take to create clearer guidelines for medical cannabis use?
21	Would pilot programs be beneficial for integrating medical cannabis into mainstream healthcare? Why or why not?
22	How can healthcare professionals collaborate with regulatory bodies to improve access to medical cannabis?
23	How can medical cannabis be effectively distinguished from recreational cannabis?
24	What communication strategies would help increase trust and acceptance of medical cannabis?
25	Do you think digital platforms and social media can play a role in medical cannabis education? Why or why not?
26	How important are standardized dosing guidelines in building trust among healthcare providers and patients?
27	What additional resources or tools would help you feel more confident in recommending medical cannabis?
28	Do you have any final thoughts on medical cannabis as a therapeutic option?
29	What do you think the future of medical cannabis looks like?
30	Is there anything else you would like to share regarding medical cannabis?

## Data Availability

The data that support the findings of this study are available from the corresponding author upon reasonable request.
